# Positive Outcomes of Early Task-Specific Training and Action Observation Mirror Therapy Following Infarction of Hand Knob Area: A Case Report

**DOI:** 10.7759/cureus.29819

**Published:** 2022-10-01

**Authors:** Moli Jain, Pallavi Harjpal, Rakesh K Kovela, Vishnu Vardhan

**Affiliations:** 1 Department of Physiotherapy, Ravi Nair Physiotherapy College, Datta Meghe Institute of Medical Sciences, Wardha, IND; 2 Department of Physiotherapy, Nitte Institute of Physiotherapy, Nitte (Deemed to be University), Mangalore, IND; 3 Department of Cardiorespiratory Physiotherapy, Ravi Nair Physiotherapy College, Datta Meghe Institute of Medical Sciences, Wardha, IND

**Keywords:** neuro physiotherapy, stroke, hand motor cortex, pseudoperipheral palsy, hand rehabilitation

## Abstract

Traumatic nerve damage or compression neuropathy is the most common cause of unilateral weakness in an upper extremity. Rarely, a central nervous system lesion may cause syndromes that initially appear to indicate peripheral nerve injury. The most well known of these is pseudoperipheral hand palsy, which is typically brought on by a minor brain lesion in the contralateral motor cortex. The term "hand knob" refers to a restricted area in the posterior frontal lobe's precentral gyrus, a lesion that causes isolated weakness in the upper extremity that mimics injury to peripheral nerves. The majority of the time, an embolic infarction is the cause of this uncommon condition. We present a case of a 52-year-old female who presented to the medicine outpatient department with sudden onset of acute pure motor paresis of the right hand. She also complained of clumsiness in her hand and several episodes of generalized headache in the parieto-occipital region in the past two weeks. Following an initial evaluation by a physician, she was advised for MRI of the head and neck, which reported occlusion in the petrous part of the left internal carotid artery, and referred to physiotherapy for further management. She was assessed and a task-specific training protocol was made for the patient, which also included physiotherapy techniques like grip exercises, mirror therapy, and strengthening. There was an improvement in the patient within a few weeks that was evident with the outcome measures post-rehabilitation. This case study is really a good case not only in regards to peculiarities and clinical presentation but also in promoting rarely implementation of rehabilitation to get patients back to their previous functional status.

## Introduction

Rarely do injuries of the central nervous system cause a single hand to become feeble. The clinical indications in such circumstances are caused by lesions of the motor cortex or the downstream corticospinal tract [1]. The "hand knob" area, which is a constrained area of the motor cortex in the precentral gyrus of the posterior frontal lobe, is where lesions cause isolated upper extremity weakness on the opposite side that mimics injury to peripheral nerves. This unusual symptom is often connected to cerebral infarction. The lesion has deceptive symptomatology that raises the possibility of peripheral neuropathy, which could make the differential diagnosis difficult [2].

This shows that patients with localized hand paresis need a thorough neurological examination and how a selective lesion in the central hand control network could be mistaken for a peripheral nerve lesion. Isolated pure motor distal hand palsy should not be confused for a peripheral nerve lesion from the perspective of a pattern-recognition heuristic clinical competence, as an early diagnosis would permit proper emergent investigations and the rapid start of therapy [3]. Isolated distal weakness in the upper extremity raises the diagnosis of peripheral neuropathy as such presentations are uncommon with the central lesion where the patient presents with hemiparesis or plegia contralateral to the lesion.

## Case presentation

Patient information

A 52-year-old female visited the neurology outpatient department (OPD) with complaints of sudden onset, non-progressive weakness of the right distal hand, early in the morning in the form of difficulty in opening the door, combing hair, and lifting objects for three days. She also complained of clumsiness in her hand and several episodes of generalized headache in the parieto-occipital region in the past two weeks. She had a significant history of hypertension for two years and was under anti-hypertensive medication with no relevant family history. There was a similar episode of sudden weakness of the right hand six months back which lasted for 10 minutes. It was diagnosed as a transient ischemic attack (TIA), which had no secondary symptoms. Following an initial evaluation, she was advised for MRI and referred to physiotherapy for further evaluation and management.

Clinical findings

The patient was alert, cooperative, and oriented to the name, place, and time of the evaluation. Following examination, she was medically alert, hemodynamically healthy, and competent upon review with blood pressure, 129/89 mm Hg; heart rate, 92 beats/min; respiratory rate, 14 breaths/ min; the temperature of 36.7°C; and oxygen saturation, 98% on room air. A neurological examination was performed. The reflexes were normal and no tonal abnormality was found. It also revealed a pure motor paresis of all flexor and extensor muscles of the right wrist and hand with a muscle strength grading of 1/5 medical research council (MRC) scale. There was no sensory disturbance present. She had difficulty with fine and gross motor movement and reduced grip strength. The remainder of her neurological and physical examination findings were within normal limits. The timeline of the current episode of the patient is represented in Table 1.

**Table 1:** Shows the timeline of the patient episode of care

S.No.	Date of events	Consultation	Findings	Suggestions
1.	18/04/2022	Medicine OPD	MRI findings reveal occlusion in the petrous part of the left internal carotid artery.	Anti-hypertensive and anti coagulants drugs
2.	21/04/2022	Physiotherapy OPD	Acute right-hand pure motor paresis	Active assisted range of motion exercises and mirror therapy in the first week. Task-specific training in the second week
3.	05/05/2022	Discharge	Improvement in gross and fine motor activity of hand	Home exercise program with strengthening exercises
4.	18/05/2022	Follow Up	She was able to perform activities of daily living independently.	Fine motor training continued. Advise the patient to be as active as possible.

Diagnostic assessment and interpretation

**Figure 1:**
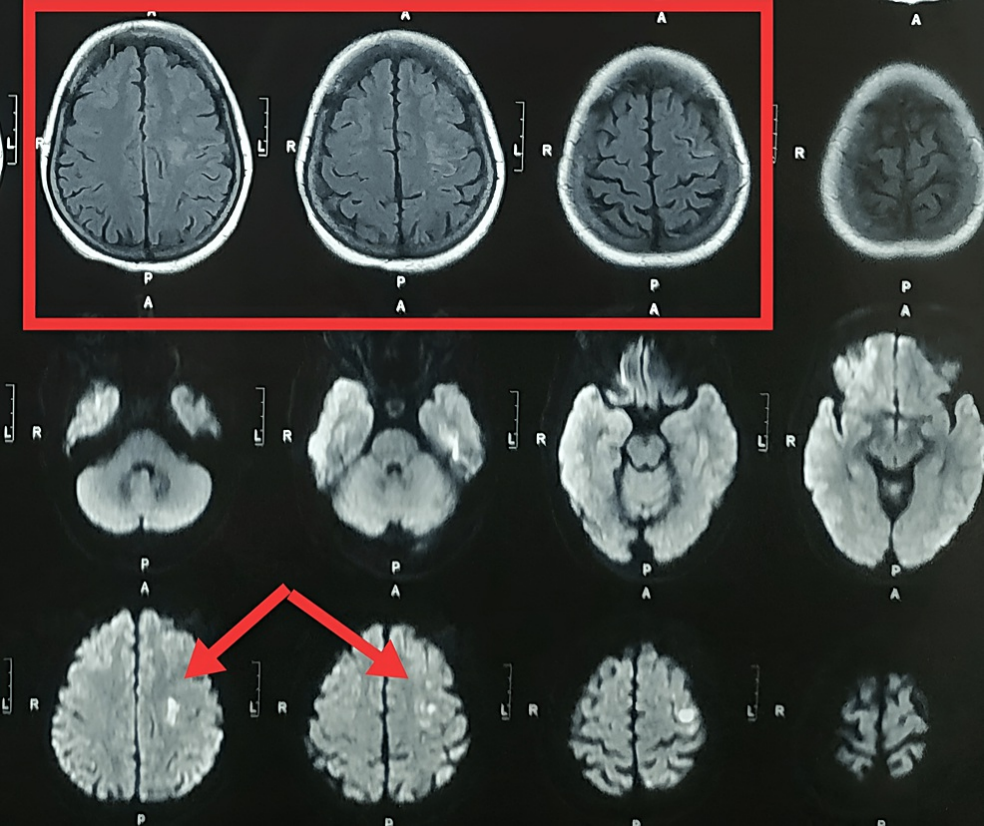
MRI of the brain Red arrow: Indicates high-intensity lesion noted at the hand motor cortex region of the left cerebral cortex. Red box: Indicates hand knob sign.

She underwent magnetic resonance imaging (Figure 1) for the brain and was found to have an acute infarct in the left high frontal, centrum semi-oval, and high-intensity lesion noted at the hand motor cortex region of the left cerebral cortex (red arrow). The hand knob sign was present (indicated in the red box). The rest of the lab investigations were within normal limits. 

Therapeutic interventions

**Figure 2:**
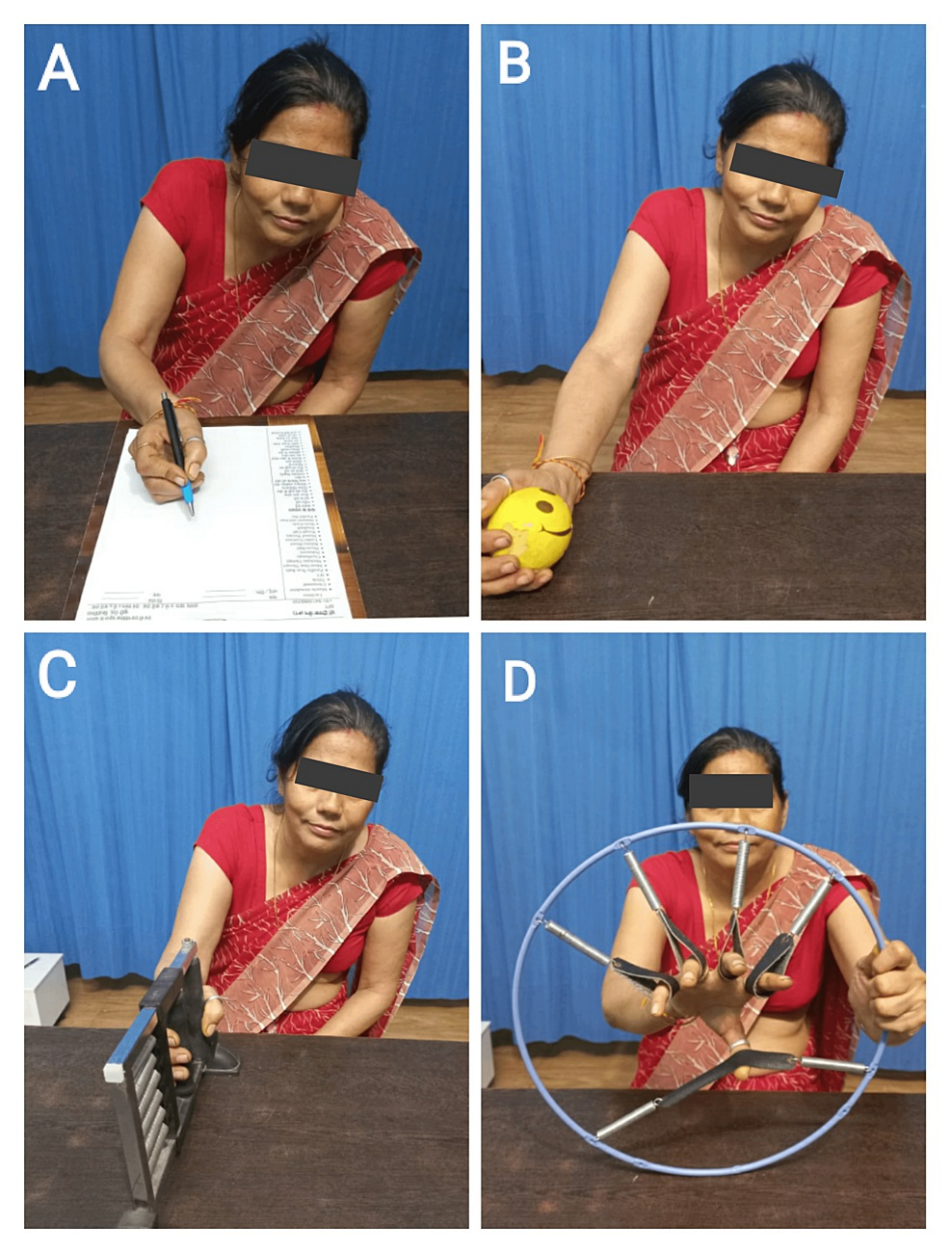
Patient performing physiotherapy intervention A: Handwriting practice. B: Strengthening with stress ball. C: Strengthening with hand gripper. D: Strengthening with finger resistance bands.

**Figure 3:**
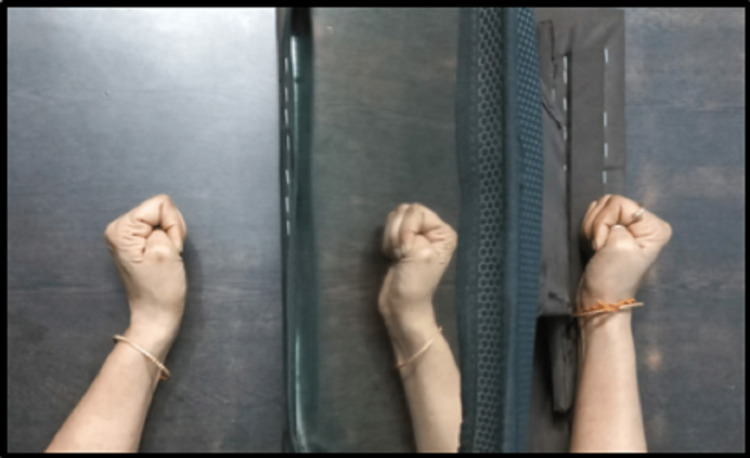
Patient performing action observation mirror therapy

Post rehabilitation patient wanted to resume her self-care activities (brushing, eating, dressing, and bathing) independently with less difficulty. Considering this, the physiotherapist’s goal was to explain to the patient importance of adherence to exercise and the importance of prescribed techniques and strategies which can be achieved through proper patient education. The long-term goals were to improve strength and fine and gross motor activities of the hand and prevent further secondary complications and rarely return to previous functional status. Table 2, Figure 2, and Figure 3 indicate the physiotherapy intervention that the patient received.

**Table 2:** Shows therapeutic intervention

Problems identified	Goal framed	Physiotherapy intervention
Decreased ability to perform hand functions	To improve the tone, thus improving functional ability	Active assisted hand movements with mirror therapy, i.e., action observation mirror therapy (Figure 3)
Fingers held in flexion	Maintaining the stability of fingers and wrist	Sitting with extended arm support, fingers, and handheld in extension with sandbag support during rest, slow sustained stretching
Decrease right-hand grip strength	To improve strength	Grip and prehension training using finger resistance bands (Figure 2D), hand gripper (Figure 2C), and stress balls (Figure 2B)
Difficulty performing gross and fine motor activities	Retraining hand function	Task-specific training and Handwriting practice (Figure 2A)
Presence of accessory shoulder movement	Avoidance of compensatory movements	Training voluntary inhibition
Decreased activity in daily living	Advise the patient to be as active as possible	Encouraged how to use the extremities to involve in activities of daily living

Follow-up and outcomes of interventions

After two weeks of rehabilitation, there was a tremendous improvement in the MRC grading for the wrist and hand flexors and extensors, i.e., grade 4, except for the ring and little finger flexors and thumb abductors, where the grade was 3. There was a regular follow-up taken for two weeks; the patient was asked to visit once a week. During the follow-up period, she was taught the home exercise program. The follow-up and outcomes are shown in Table 3.

**Table 3:** Shows pre- and post-rehabilitation outcomes

Outcome measure	Pre Rehabilitation	Post Rehabilitation	Follow up
Duruoz hand index	74	25	12
Upper Extremity Function Test (affected hand)	21 (trace)	88 (functional)	96 (maximum function)

## Discussion

A lesion limited to the central nervous system is rarely the source of isolated upper extremity weakness as the clinical symptoms point to a peripheral neuronal illness. Cortical hand knob accounts for less than 1% of all hospitalized ischemic strokes in single-center survey [2]. Nerve conduction investigations are the standard first step in the workup of such individuals [4]. But, when electrophysiological test results are normal, another explanation should be investigated. When the precentral gyrus (primary motor cortex) is injured, clinical symptoms that resemble peripheral nerve lesions are produced. This condition is known as pseudoperipheral palsy [2,5,6]. When examining peripheral motor nerve deficits in the presence of normal nerve conduction velocities, vascular injury in the hand knob area should be taken into account [7]. According to research on eight patients, distinct cerebral infarction in the precentral gyrus was the cause of their isolated hand palsy [8]. There have been reports of isolated hand palsies caused by embolic strokes involving the hand knob area, large atherosclerotic infarctions of the cerebral cortex's vascular border zones, subcortical lacunar infarctions, and, in rare cases, infarctions of the inferior parietal lobe brought on by severe carotid stenosis or dissection [9,10].

In the current case report, acute infarct in the left high frontal, centrum semi-oval, and occipital regions with occlusion in the petrous part of the left internal carotid artery may have caused the isolated hand paralysis in the motor homunculus region of the left precentral gyrus. The clinical characteristics and normal nerve conduction velocity results ruled out the likelihood of peripheral nerve damage. Making that distinction requires careful evaluation of the patient's history, physical examination, time of onset, and assessment of their risk factors for cerebral vascular accidents. Most significantly, just being aware that this entity even exists can assist in avoiding misdiagnosis as a peripheral lesion and direct the patient toward the right kind of care. It is vital to be aware of the extensive differential diagnoses of isolated hand weakness since ischemic stroke should also be taken into account as a possibility for the rare cause of isolated hand palsy.

From the very first day of physiotherapy, action observation mirror therapy (30 minutes of self-directed mirror therapy five times/ week) was started and progressed to task-specific training by the next week. The treatment protocols were in line with a previous study suggesting improvements in the hand function and handwriting skills of patients through task-oriented training and mirror therapy [11,12]. This also led to a reduction in the impairment level of the upper extremity. The patient was urged to carry out as many effective repetitions as she could. Although completing the tasks was the main focus, the therapist designed each exercise to minimize the usage of compensatory mechanisms, i.e., flexor synergy of wrist and hand. The patient was provided regular feedback with knowledge of performance and knowledge of results during training. Prior research has shown that externally focused feedback improves motor performance in various contexts and also contributes to the automation of skills [13]. 

Patient satisfaction seems to be closely related to a successful rehabilitation outcome. Due in large part, we believe, to the type of treatment approach and techniques that emphasized task-specific practice and avoided compensatory movement throughout the sessions, our patient responded to treatment extremely well and her compliance to sessions was up to the mark. We also credit the reliability of the home program for contributing to our success. Week after week, we saw consistent development.

## Conclusions

Such cases demonstrate the utility of precise neurological examination with patients having central lesions presenting with pure central hand and finger weakness to rule out more peripheral lesions. The clinical viewpoint helps in timely diagnosis, emergent investigations, and implementation of early intervention to promote better patient outcomes. This case study is uncommon not only in regard to peculiarities and clinical presentation but also in promoting rare implementation of rehabilitation to get patients back to their previous functional status.
